# Supplementary material for: Patterns of Intron Gain and Loss in Fungi
Source: PLoS Biol. 2004 Nov 30;2(12):e422. doi: 10.1371/journal.pbio.0020422 (PMC532390; doi:10.1371/journal.pbio.0020422)
Supplement: Table S1 — Also available at http://genes.mit.edu/NielsenEtAl/. (4.3 MB ZIP). [file pbio.0020422.st001.zip › NielsenEtAl/html/476.html]

AN0416.1.NCU00899.1.MG04519.1.FG00714.1


```
 CLUSTAL W (1.82) Multiple Sequence Alignments - Introns Inserted


Sequence 1: MG04519.1	332 aa
Sequence 2: FG00714.1	427 aa
Sequence 3: NCU00899.1	360 aa
Sequence 4: AN0416.1	335 aa
Alignment Length: 430 aa
Number Identitical Residues: 125 aa
Alignment Score (without introns) 6368


MG04519.1 	------------------------------------------------MASAIFRNY---
NCU00899.1	MAQNIASRPK------------------KGVFDFLESRWAKFFFAIVALQAIIGVTF---
FG00714.1 	MTTRLDSRKSLLGADNGYGYGHSYNNSYDNSYAPSPVPSPQPIPQPPAFQAAVEPTYNQG
AN0416.1  	-------------------------------MYKPNSLWTWSFAIVTLVQTVITLAL---
          	                                       .        . : :       

MG04519.1 	---------R~FPG~HHMSDIRI0NSVK-----------------------~--------
NCU00899.1	--------ES2YIF~GRFQDSLE~DYTT-----------------------~--------
FG00714.1 	FAYREGLTPS~VPS~QSMKNGKI~DFMKSRWPACFMAVSLLQAILCICFEA2YIFGKFQV
AN0416.1  	----------~-QC2YIFANFQL~QLTA-----------------------~--------
          	                 : :    :                                   

MG04519.1 	--GDQVSDEAIEGAPLRSIPTFLTLFIFGFLYMLVLVWDALRMKNTIQVIGVCIANLAMM
NCU00899.1	------GENEERDAQYMTIPTFLALFIFGFLYVLFLTWDALRWKNTIQIIGLCIANLALF
FG00714.1 	NLGTHNPDPEVQ-SQYKTIPTFLTLFIFGFLYTLVVVWDALRQKNTIQIIGVCFSNLALM
AN0416.1  	--------EAELVTSSKTIPTFLALYSFGFLYELFLVYDALRLKNTIQVIGLCLCNIGLL
          	             :   :*****:*: ***** *.:.:**** *****:**:*:.*:.::

MG04519.1 	VYTAIQIDQIHDAIVLLENAGILLTTT--KELWDDCRPLLFTIPVIVSVCTLGMAFISWK
NCU00899.1	VYTILQIDQIDRSISKLQLAGIFRPLEVHEAVWHACAPFLYTIPPVIGVATVAMSACAWQ
FG00714.1 	VYTTIQVDQIKEAFDILGEHSALRPEMPATELWHDVKPYLVAIPAIIALATLVMGFLSWK
AN0416.1  	VYGAVQVQQIKEAVTVLTDN-----DAVNPDIWAETQPFLIIIPIVVAMGSVLMMIVAWK
          	**  :*::**. :.  *              :*    * *  ** ::.: :: *   :*:

MG04519.1 	LYQEFAWDILKQIGADYRMKRRFLHYQ0IYIALLKFDFFFFLGFTVQFLVVVRDKTTDTE
NCU00899.1	LYQEFAWDILKQIGADYRMKKRFLHYQ0IFIALLKFDFFFFLGFTIQFLVVVTGKT-SIE
FG00714.1 	IYQEYAWDILKNIGADYRMKKRFLHYQ0IYIALLKFDFFFFLGFIVQFVVVVADKD-DPE
AN0416.1  	LYDEFAWSIYKHISADLRMKRRYLTYQ0IYIALLKFDLFFFLGFTIQFLVIVTNRK-DTE
          	:*:*:**.* *:*.** ***:*:* ** *:*******:****** :**:*:* .:  . *

MG04519.1 	FRLTLVAIPVTIAILLFAAFFVRRENKIGMSAII~VLYFGALSYFFFKLVRIYQPGHKEK
NCU00899.1	LGLTAAAIPVTIIILLCAAFFTQRENRVGMYAVI~LLFFGGLAYFLFKLVRIYSKSHGWH
FG00714.1 	FALTIVTIPITILILLAAAFFTRRENKAGMIAIV~VLYLGGLSYFIFKLVRIYQPGHKQN
AN0416.1  	FALTIAAIPVTILILLCAALFVKRESSIGMIAII0LLYFAAMAYFLFKLVRMYQPATAND
          	: ** .:**:** *** **:*.:**.  ** *:: :*::..::**:*****:*. .   .

MG04519.1 	YQAAARSLTAFAVLTIILIILTITNAFICMNNFGAGLRNHLRSPSRD---EEKPDATSSI
NCU00899.1	YTPVKRSLTAFAVMTILLILITIANAFICTSNFGNGLKTHLVKPQGRD--PEKDDIN-SF
FG00714.1 	YEAVRRSLTAFAVITILLIILTIINAIVCMLNFGKGLKPHLQALSRKRKVEEKPDVN-SI
AN0416.1  	YSTARRSLTFFAIITLILIVMTIINACVCMHNFHRGLKPHINKKKKG-----KDGEKGST
          	* .. **** **::*::**::** ** :*  **  **: *:   .       * . ..* 

MG04519.1 	SMHDVKPQLPSRMTID
NCU00899.1	QMTDQKPKLTTRMTID
FG00714.1 	NMQDVKPQIPSRMTID
AN0416.1  	ELSNVAGQVPTRMMID
          	.: :   ::.:** **
```
